# A Peculiar Symptom of Cocaine

**Published:** 1888-11

**Authors:** Wm. Jefferson Guernsey


### A PECULIAR SYMPTOM OF COCAINE.

Editors Homœopathic Physician:—The following from an allopath shows a keener scent than some of our *demi-semi* homœopaths possess.

The symptom is a *peculiar* one, therefore important.

Yours,
Wm. Jefferson Guernsey.

Schadle (J. E.) on a Peculiar Effect of Cocaine.—Preparatory to an intranasal operation, pledgets of cotton saturated with a four per cent. solution of Cocaine were introduced into the nostrils on several occasions. On each application the patient, a man of thirty-five, mentioned a cold, "gone," relaxed feeling about the external genitals and a *sensation as if the penis were absent*. Toward the end of treatment he noticed a permanent weakness of the sexual organs, and finally seminal losses and impotence set in and continued until the Cocaine was entirely withheld.—*Medical Register*, August 11th, 1888.

